# Unite and Co-Operate

**Published:** 1887-02-19

**Authors:** 


### UNITE AND CO-OPERATE.

Unite and co-operate is the message which the Jubilee Year brings to the medical charities throughout the country. In no part of the empire is the necessity for union and co-operation more paramount than in London. In the metropolis the hospitals have sprung up without any definite regard to the requirements of population or the growth of individual communities, with the result that there is a tendency for the work of each institution to be overlapped by that of its neighbour. For the same reason, impostors and improper cases of all kinds are enabled to go from hospital to hospital until they obtain what they require. By united action and earnest co-operation the relief of sickness will be extended and economised to an extent difficult to assess. The present hotch-potch system of raising income and distributing medical relief tends to increase the difficulties of all the hospitals, by giving the ungenerous the maximum of opportunity for criticism and complaint. No one disputes these patent facts; but to the present day united action and earnest co-operation amongst managers of the London medical charities have been conspicuous by their absence.

Will the message of the Jubilee Year be taken to heart, and so produce good fruit? There are encouraging signs that it will. Last week the Lord Mayor presided over a large meeting at the Mansion House, which was attended by the managers of all the principal hospitals in London, many representative men being present, at which a determination to unite and co-operate was very generally expressed. Last year the Hospitals Week movement included the division of London into six districts, in each of which a public meeting was held, at which eminent speakers attended, including the Duke of Cambridge and the Prime Minister, Lord Salisbury. It was urged with truth at the time, that these meetings were not numerous enough to cover so vast an area as that included in Greater London. This year it has been decided to have twelve district meetings in the week commencing June 6th, and six large central meetings in the following week, constituting, as heretofore, The Hospitals Week, and ending with Hospital Sunday, June 19th. The presidents and speakers of the twelve district meetings will be selected from the public men who are well known to the residents in each locality. The six central meetings will be presided over by the Duke of Cambridge, the Lord Mayor, and four other eminent men, whilst the Prime Minister, Lord Salisbury, has already intimated his intention to be present and speak at the meeting at the Mansion House. The clergy and ministers of religion are showing a genuine interest in the movement, and we venture to hope that this year the present Archbishop of Canterbury, who has never shown any public interest in our hospitals, will make a point of being present at one of the meetings, and thus show to those who assert to the contrary, that he is as warmly interested in the maintenance of hospitals as all the other leaders of religious thought in this country have proved themselves to be over and over again. There can be no doubt, from the experience of last year, that awakened and earnest feeling on the part of the laity in support of the hospitals must act as a healthy stimulus upon the clergy and ministers of religion, who have heretofore had to bear the heat and burden of the organisation of Hospital Sunday without the hearty co-operation of all classes of the community. To all who have preached on behalf of the hospitals, hearty thanks are abundantly due, and we believe the present awakening of the people to the importance of adequately supporting hospitals must be genuinely welcomed by the ministers of all denominations.

It was satisfactory to note at the Mansion House Meeting of hospital representatives that hospital managers are beginning to realise that some change in their methods of raising income must be resorted to if the voluntary principle of supporting hospitals is to be maintained in this country. London, for the purposes of raising voluntary contributions in support of hospitals, may be compared to a vast area of unreclaimed land in a distant colony of the empire. The part of most colonies under cultivation relatively to the whole area is remarkably small. So the number of persons in London who give anything at all to the hospitals is relatively insignificant. In the Colonies additional labour is welcome, because it means the increase of the area of civilisation, and the consequent development of the resources, not only of the colony proper, but of each individual inhabitant. So in London The Hospitals Week last year, by extending the area of interest in the voluntary support of hospitals, resulted in an increase of the contributions to individual institutions by Londoners. In one instance a London hospital secretary, who recognised this probable result of the new movement by issuing 1,000 circulars in the fortnight following Hospital Sunday, raised £1,000 in the whole. That is, he received £1 for each circular letter of appeal which was issued from his office. These facts are becoming more and more recognised, and they should combine to unite hospital managers in one enthusiastic effort in this year of Jubilee, to raise at least £100,000 on Hospital Sunday 1887. No effort will be spared by the Lord Mayor, or the Council of the Metropolitan Hospital Sunday Fund, to bring about this desirable result, and we can only hope that the Chairmen and Committees of the London hospitals, with their secretaries and officials, will decide to heartily co-operate with the central organisation at the Mansion House, and so secure the establishment of the voluntary principle of hospital support upon a permanent and sound basis.

